# First year of in-house forensic neuropathology consultations in Helsinki, Finland

**DOI:** 10.1007/s00414-024-03399-6

**Published:** 2025-01-13

**Authors:** Petteri Oura, Hilla Mäkinen, Roosa Ruotsalainen, Miko Ruokomäki, Antti Virtanen, Antti J. Hakkarainen

**Affiliations:** 1 Forensic Medicine Unit, Finnish Institute for Health and Welfare, P.O. Box 30, Helsinki, FIN-00271 Finland; 2 Department of Forensic Medicine, Faculty of Medicine, University of Helsinki, P.O. Box 21, Helsinki, FIN- 00014 Finland

**Keywords:** Central nervous system, Legal medicine, Medico-legal autopsy, Pilot, Cost, Outsourcing

## Abstract

In July 2023, an in-house forensic neuropathology consultation pilot was established at the Helsinki office of the Forensic Medicine Unit, Finnish Institute for Health and Welfare. This offered an alternative to the previous practice of full outsourcing to a hospital neuropathology department. This paper aims to introduce the first year experiences of the pilot. The in-house consultant team comprised two forensic pathologists with special training in neuropathology. In-house consultations were offered for medico-legal autopsy cases with traumatic brain injury (TBI) and hypoxic-ischaemic encephalopathy. The in-house histology laboratory participated in the pilot, implementing beta-amyloid precursor protein (β-APP) stain at the end of the pilot year. An electronic database was also developed for data collection and reporting. The characteristics, costs, and duration of the first 25 consultation cases were reviewed. The cases involved mostly male decedents (84.0%) with TBI (84.0%). The median total cost of a pilot case was €624, which was substantially lower compared to the previous outsourced practice (€1013 per case + €38/84 for each microscope slide with special/immunohistochemical stain, respectively). After the implementation of β-APP stain into the in-house laboratory service, the median total cost of a pilot case was reduced further to €94. The median duration of an in-house consultation was 2.3 months. Thanks to a favourable in-house atmosphere, the first year experiences are encouraging. While the costs of the in-house consultation practice appear to be markedly lower than those of an external provider, both alternatives should be available for cases where sufficient expertise cannot be found in-house.

**Supplementary Information:**

The online version contains supplementary material available at 10.1007/s00414-024-03399-6.

## Introduction

Neuropathological expertise is of paramount importance in the medico-legal cause-of-death investigation practice [1–3]. As this expertise may lie outside the scope of a general forensic pathologist, proficient consultation opportunities with a neuropathology expert may play an essential role in the process. While some institutions have a neuropathologist readily under their authority, others need to acquire this expertise from an external provider. Even though the consultation practice can be organized in very different ways depending on the institution, there is a paucity of previous data on the usage, benefit, and costs of neuropathology consultations in medico-legal autopsies [4, 5].

In Finland, medico-legal autopsies are performed by forensic pathologists at the regional offices of the governmental forensic medicine authority (Forensic Medicine Unit, Finnish Institute for Health and Welfare). Of a total of five regional offices, the Helsinki office is the largest one, comprising approximately 10 forensic pathologists, 3000 autopsies, and 30 neuropathology consultations per year. Previously, consultations requiring neuropathological expertise have been fully outsourced to an external provider. In July 2023, an in-house neuropathology consultation pilot was established as an alternative to the previous practice. The aim of this paper is to introduce the concept and first year experiences of the pilot.

## Materials and methods

### Previous practice

In Finland, a death needs to be reported to the police if there is a suspicion of homicide, suicide, accidental death, medical/surgical adverse event, occupational disease, or if the death is sudden and unexpected [6]. In most cases, the police orders a medico-legal autopsy to be performed by the governmental forensic medicine authority (Forensic Medicine Unit, Finnish Institute for Health and Welfare). Currently, the medico-legal autopsy rate is approximately 15% of all deaths.

**Fig. 1:**
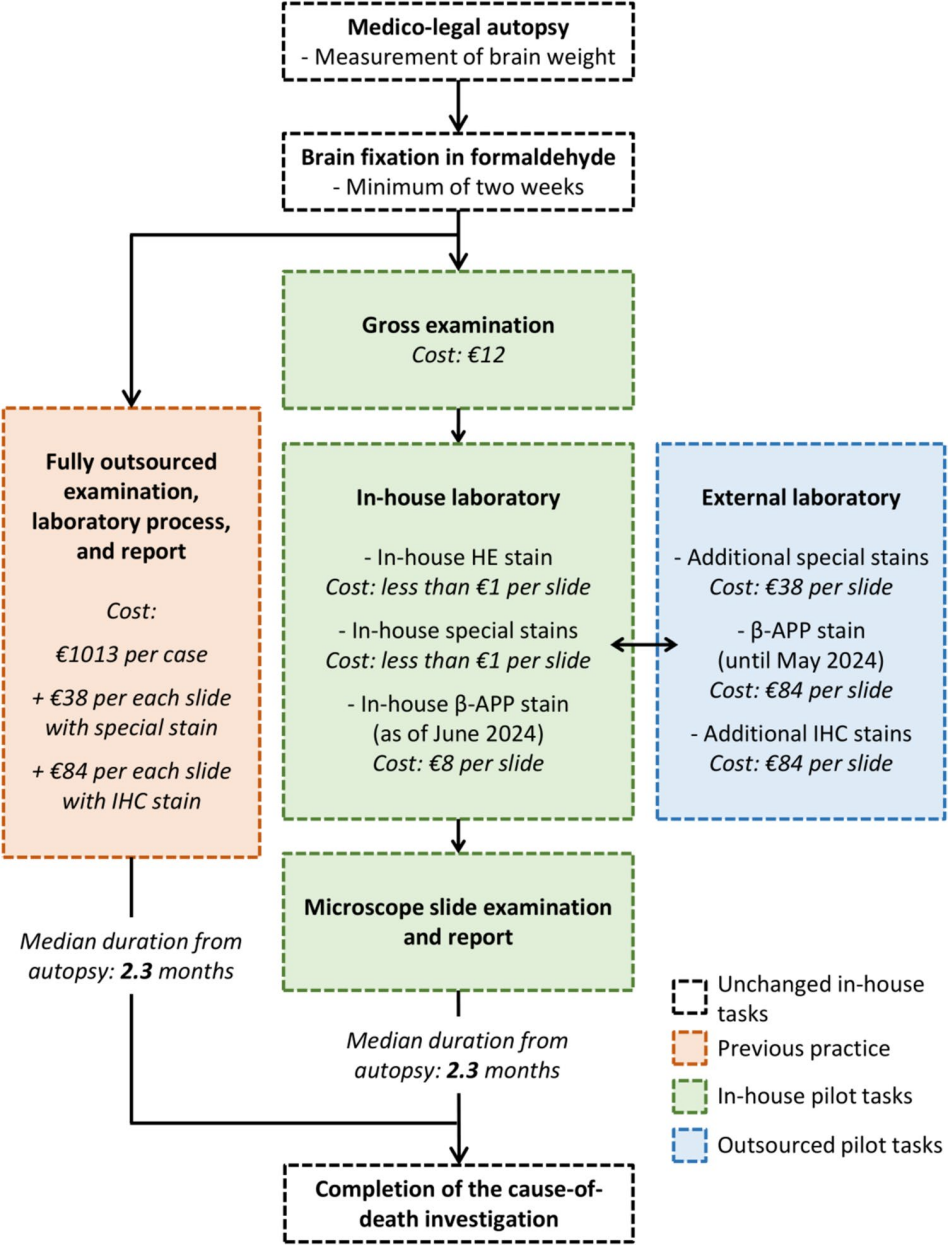
Flow chart of the forensic neuropathology consultation process illustrating both previous outsourced practice and in-house consultation pilot. β-APP = Beta-amyloid precursor protein, HE = Hematoxylin-eosin, IHC = Immunohistochemistry

Previously, all neuropathology consultations at the Helsinki office of the Forensic Medicine Unit were outsourced to a large pathology department of a tertiary-level hospital (Fig. 1). The formaldehyde-fixed brain and other possible central nervous system (CNS) samples of the medico-legal autopsy cases that required a neuropathology consultation were delivered to the external provider. Gross examination, laboratory work, and microscopic examination of the samples were performed by a hospital neuropathologist.

Over the recent years, the external neuropathology consultation rate has been 1.4%, corresponding to one consultation per 70 medico-legal autopsies [5]. The main consultation theme has been traumatic brain injury (TBI); the consultant neuropathologist has often been asked to evaluate only the presence of diffuse traumatic axonal injury (dTAI) in the immunohistochemical (IHC) β-amyloid precursor protein (β-APP) stain.

In 2024, the cost of an external neuropathology consultation was €1013, plus an additional cost of €38 for each microscope slide with a special stain (such as Prussian blue or Congo red), plus an additional cost of €84 for each microscope slide with an IHC stain (Fig. 1). The median time from autopsy to a completed neuropathologist’s consultation report was 2.3 (interquartile range [IQR] 1.4—3.5) months.

### Overview of the pilot

In the spring of 2023, a team of two in-house neuropathology consultants was compiled within the Helsinki office of the Forensic Medicine Unit, consisting of a neuropathology-oriented forensic pathology resident (PO) and board-certified forensic pathologist (AJH). The in-house team developed a framework and protocol for the pilot together with the forensic pathology team leader of the office (AV). As the initial step, the team systematically reviewed all external neuropathology consultation cases from the previous seven years; a detailed analysis will be published separately.

**Fig. 2:**
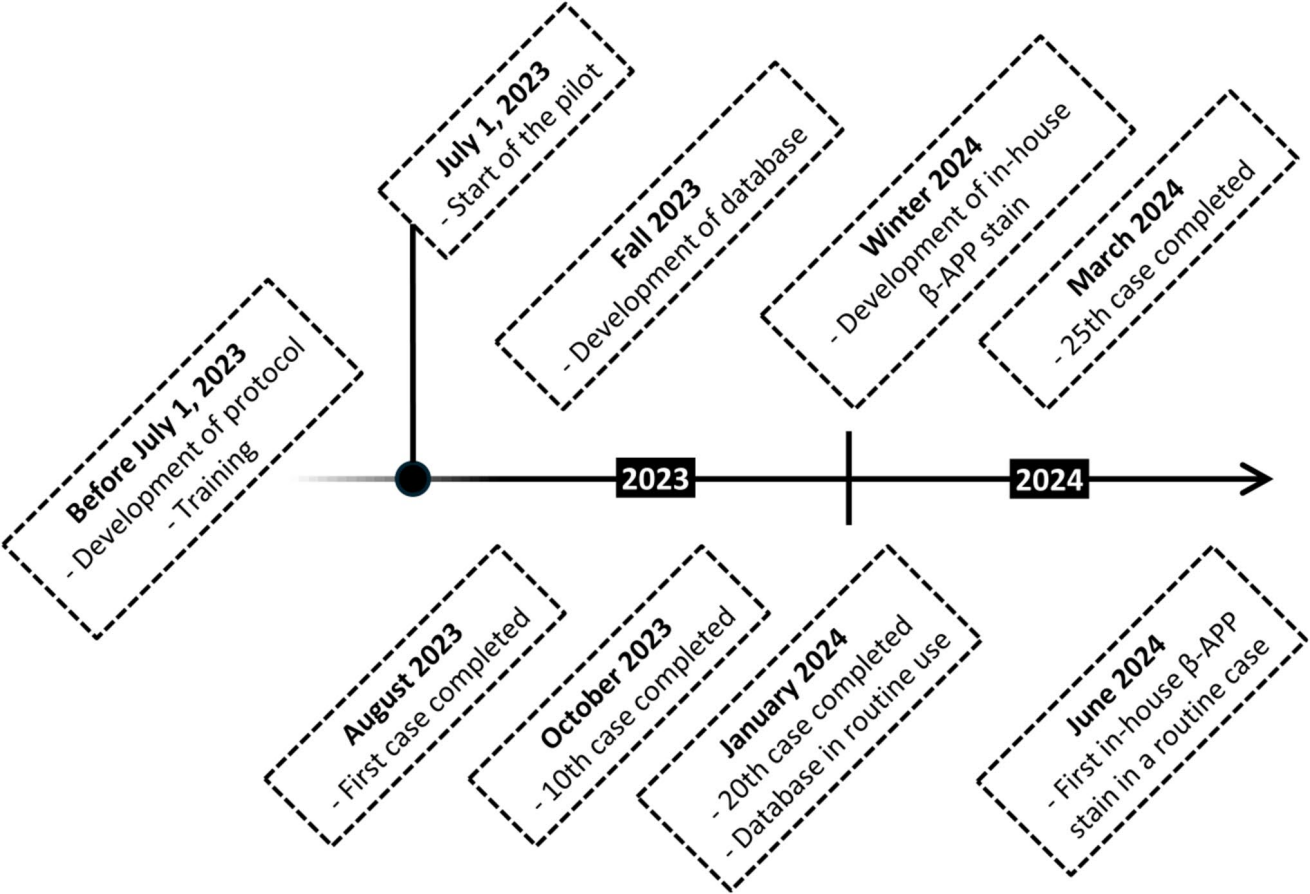
Timeline demonstrating the milestones of the first year of the in-house forensic neuropathology consultation pilot. β-APP = Beta-amyloid precursor protein

The pilot commenced in July 2023. Forensic pathologists performing routine autopsies at the Helsinki office were informed of the possibility to refer cases to the in-house consultation team. They had, however, the autonomy to refer any case to the external neuropathologist, as before, if they found it necessary. A flowchart of the pilot is included in Fig. 1. The milestones of the pilot’s first year are summarized in Fig. 2.

Although the project was primarily conducted as operational development, the cases were licensed for scientific use by research permits from the Finnish Institute for Health and Welfare (THL/1802/6.02.00/2023, signature dates 2023-04-21 and 2024-02-20). This study was performed in accordance with the Declaration of Helsinki and national legislation on medical research. As the collection of data for scientific use was retrospective and register-based, ethical approval was not required.

### Training

During the years leading up to the pilot, both members of the in-house team underwent several months of neuropathology training as part of their forensic pathology residency. The training was completed at a large pathology department of an academic, tertiary-level hospital; it was the same one that provides external neuropathology consultations for the medico-legal cases. Training involved the examination of both surgical and autopsy-derived CNS samples under the supervision of board-certified neuropathologists. In addition, a member of the in-house team (PO) participated in a forensic neuropathology intensive course organized by the European Confederation of Neuropathological Societies (Euro-CNS) in 2023.

### Case selection

At first, in-house consultations were offered for adult TBI cases that involved only the assessment of dTAI. After the first few cases, the inclusion criteria were extended to cover all TBI and hypoxic-ischaemic encephalopathy cases. In contrast, paediatric cases as well as those in which the consultation question addressed the diagnosis of a CNS disease (e.g., demyelinating disease or tumour) were referred to the external neuropathologist, as before.

### Neuropathological examination and sampling protocol

As part of the routine medico-legal autopsy, the fresh brain was examined from the outer surfaces and its weight was recorded by the referring forensic pathologist. Once the brain had been suspended in a 10 L bucket containing 10% formaldehyde for a minimum of two weeks, the gross examination was performed by a member of the in-house consultation team. An assistant (HM) was often present to aid with documentation and photography, and also the referring forensic pathologist was offered the possibility to participate in the examination.

In the gross examination, first, the outer surfaces of the formaldehyde-fixed brain were inspected, and the major arteries were dissected. Next, the brainstem and cerebellum were excised from the cerebrum at the level below the mamillary processes, and the brainstem was separated from the cerebellum at the level of the cerebellar peduncles. The brainstem and cerebellum were cut in axial and sagittal slices, respectively, of approximately 0.5 cm in thickness. Then, the cerebrum was divided into an anterior and posterior half by a coronal cut aligned with the mamillary bodies. The anterior and posterior halves were cut into approximately 1 cm thick coronal slices with the help of a standardized cutting tool. Finally, all slices were placed on a board for closer inspection, photography, and sampling.

Photographs were obtained from the external surfaces of the brain (convexities and base) before cutting, as well as from the sectioned cerebrum, cerebellum, brainstem, and potential macroscopic lesions therein before sampling.

Tissue samples were routinely obtained from a total of 23 regions (Supplementary Table 1) as well as from macroscopic lesions at the discretion of the in-house consultant. The samples were placed into standard tissue cassettes with internal dimensions 2.6 × 3.0 × 0.5 cm. The routine sampling sites were selected to cover the regions relevant to the medico-legal framework, most importantly the assessment of dTAI and hypoxic-ischaemic neuronal injury, but also to cover the assessment of common neurodegenerative diseases. The consistency of documentation and sampling were the cornerstones of the protocol.

In the first year of the pilot, a typical case was estimated to consume 3–4 h of the in-house consultant’s working time (~ 1.5 h for gross examination + ~ 2 h for microscopy and report). Of particular note is the fact that the in-house team had the possibility to consult an external board-certified neuropathologist during the process at a low threshold.

### Laboratory protocol

During the first year of the pilot, tissue stains were obtained from two different parties, namely the in-house histology laboratory, and an external provider. As illustrated in Fig. 1, the in-house laboratory provided hematoxylin-eosin (HE) and certain special stains (Prussian blue or Congo red), as well as β-APP IHC as of June 2024. Other special and IHC stains, as well as β-APP until May 2024, were obtained from an external accredited laboratory at a local pathology department.

In the in-house laboratory, HE and special stains were performed on 3–6 μm sections of formaldehyde-fixed, paraffin-embedded tissue blocks, with the help of an automated slide stainer (Sakura Tissue-Tek Prisma, Sakura Finetek Europe B.V, Alphen aan den Rijn, The Netherlands). The procedures followed general guidelines for HE [7], alkaline Congo red technique [8], and Perls’ Prussian blue reaction for ferric iron [9].

The in-house β-APP protocol was as follows: One 4 μm paraffin section on a charged slide from each sample was stained for Alzheimer Precursor Protein (APP) A4. IHC staining was performed using horseradish peroxidase polymer method in a humid chamber with EnVision FLEX, High pH kit (code K8002, Dako, Glostrup, Denmark). Deparaffinization and heat-induced epitope retrieval was performed in 3-in-1 target retrieval solution, high pH (EnVision FLEX Kit) in PT Link machine (Dako, Glostrup, Denmark) for 20 min in 97 °C. After epitope retrieval, endogenous peroxidase activity was blocked using Peroxidase Blocking reagent (EnVision Kit, 5 min), followed by primary antibody (Anti-APP A4 Antibody, a.a. 66–81 of APP {N-Terminus}, clone 22C11, 1:500, 1 h RT, code MAB348, LOT 4000311; Chemicon, Merck KGaA, Darmstadt, Germany). For detection of the antibody, HRP reagent (EnVision FLEX Kit, 20 min) was applied followed by chromogen-substrate-solution (DAB+, substrate buffer, EnVision FLEX Kit, 10 min) for visualization. Mayer’s hematoxylin was applied for 3 min as a counterstain. Slides were then dehydrated through graded alcohols, cleared with xylene and coverslipped for permanent mounting.

The β-APP protocol was implemented by an in-house laboratory scientist (RR) with the help of a member of the consultant team (PO). Validation was completed by means of a head-to-head comparison of slides stained at the external accredited laboratory (“gold standard”) and at the in-house laboratory. The comparison was based on a total of ten cases, with positive and negative findings in the diagnostically relevant brain regions for axonal injury (i.e., pons, internal capsule, corpus callosum).

### Database

**Fig. 3:**
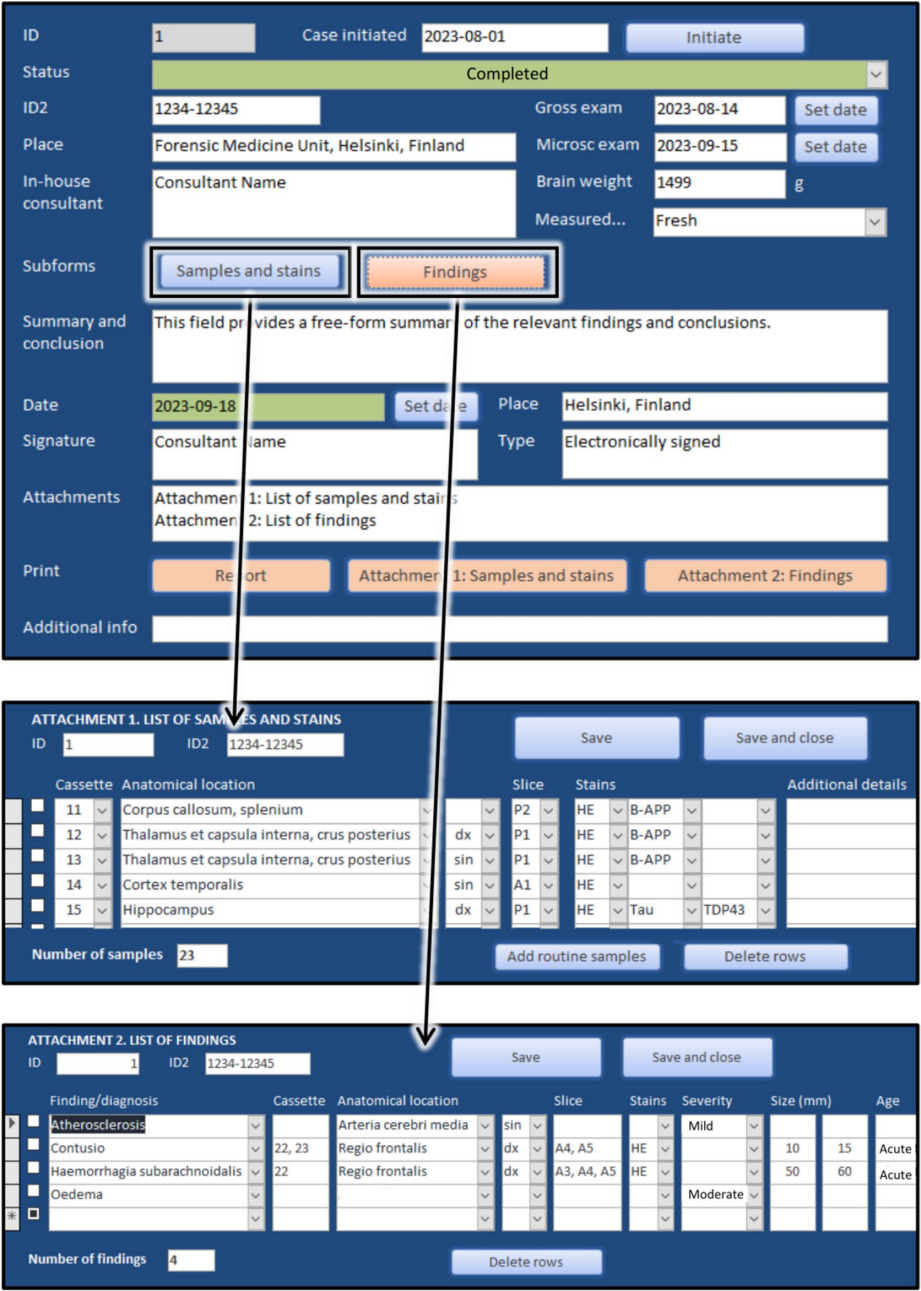
Snapshots from the interface of the electronic database that was developed for the in-house forensic neuropathology consultation pilot. Top: General view of a case. Middle: Samples and stains subform. Bottom: Findings subform

A tailored electronic database was developed by an in-house expert (MR) as a platform to prepare consultation reports and collect data for research use. It was used in all stages of the consultation and also allowed potential delays to be monitored. Most importantly, the database included details of samples and findings, most data being recorded in a structured form; a breakdown of the variables is provided in Supplementary Table 2. The database was built on Microsoft Access 2016 (Microsoft Corporation, Redmond, WA, USA) and located on a secure in-house network drive with regular back-ups (Fig. 3).

In the gross examination stage, details of samples, stains, and macroscopic findings were entered into the database. Microscopic findings were added when the microscope slides were examined, and the previous macroscopic findings were amended if necessary. Finally, the in-house consultant formulated and signed an open-ended report summarizing the relevant findings and conclusions for the referring forensic pathologist. Once the case was completed, all documents (i.e., the report accompanied by a structured list of samples and findings; photographs; and the physical microscope slides) were handed over to the referring forensic pathologist. These were later stored in the official medico-legal case files and physical microscope slide archive.

### Costs and duration

Costs were defined as additional expenses for the Forensic Medicine Unit resulting from the in-house consultation practice. These included identifiable material costs in the gross examination and laboratory stages but not personnel costs as they would have remained unchanged regardless of the pilot. It is essential to note that the cost estimates are approximate and intended to offer a general view of the cost perspective.

In the gross examination stage, the costs comprised a disposable sponge, knife blade, tissue cassettes, and formaldehyde solution, summing up to €12 per case in total (Fig. 1). As for the laboratory work, the costs depended on the number and type of stains required. HE and special stains (Prussian blue and Congo red) were performed in-house at the cost of less than €1 per slide; in cost calculations these were rounded to €1 per slide. If other special stains or IHC stains were required, they were obtained from an external provider (€38 and €84 per slide, respectively). For the first 25 pilot cases, also β-APP was acquired from an external provider, as the in-house laboratory had not yet implemented it into its range of services. The implementation was successfully completed in June 2024; after implementation, the cost of a β-APP slide reduced significantly from €84 to €8 per slide.

Duration of the consultation was defined as the difference (months) between the autopsy and: (1) gross examination of the formaldehyde-fixed brain; (2) examination of microscope slides; and (3) completion of the consultation.

### Statistical analysis

The statistical analysis was descriptive and aimed to briefly characterize the first 25 cases subjected to the in-house consultation. Key variables, extracted from the electronic database and the medico-legal case files, included the following: sex (male/female), age (years), brain weight (grams), consultation questions (TBI/hypoxic-ischaemic encephalopathy/other; multiple-choice), frequencies of samples and stains, frequencies of findings.

The distributions of categorical variables were presented using percentages (%) and frequencies (n), and those of continuous variables were presented using medians, IQRs, and full ranges (minimum—maximum). Statistical analysis was performed in IBM SPSS Statistics version 27 (IBM Corporation, Armonk, NY, USA).

## Results

###  Case characteristics

The descriptive analysis was based on the first 25 cases referred to the in-house neuropathology consultation pilot. This corresponded to an average of one case per 1—2 weeks. The majority of cases were male (84.0%) and the age range was 16—80 years (Table 1). In line with the case selection criteria, 84.0% of the consultation questions addressed TBI, and 20.0% addressed hypoxic-ischaemic encephalopathy. Of the two cases that had other consultation themes, one addressed epilepsy and the other spontaneous intracerebral haemorrhage.

**Table 1:** General characteristics of the pilot cases (*n* = 25)

Characteristic	Percentage (*n*) or Median (IQR, full range)
Sex	
Male	84.0 (21)
Female	16.0 (4)
Age (years)	42 (22–65, 16–80)
Material	
Brain	100.0 (25)
Spinal cord	12.0 (3)
Consultation theme	
Traumatic brain injury	84.0 (21)
Hypoxic-ischaemic encephalopathy	20.0 (5)
Other	8.0 (2)
Fresh brain weight (g)	1455 (1358–1527, 1017–1694)
Number of samples obtained	25 (23–26, 4–28)
Most frequent stains	
Hematoxylin-eosin	100.0 (25)
β-APP	96.0 (24)
Prussian blue	12.0 (3)
Number of stained slides requested	
Hematoxylin-eosin	25 (23–26, 4–28)
Special stains (Prussian blue, Congo red)	0 (0–0, 0–4)
β-APP	7 (4–8, 2–10)
Other immunohistochemical stains	0 (0–0, 0–6)
Most frequent findings/diagnoses	
Parenchymal haemorrhages	88.0 (22)
Oedema	64.0 (16)
Hypoxic-ischaemic neuronal injury	52.0 (13)
Vascular axonal injury in β-APP	52.0 (13)
Contusion	36.0 (9)
Atherosclerosis	32.0 (8)
Cost per case	
Gross examination, in-house	€12 (fixed cost)
Hematoxylin-eosin slides, in-house	€25 (23–26, 4–28)
Special stain slides, in-house	€0 (0–0, 0–4)
β-APP slides, external laboratory	€588 (336–630, 168–840)
* β-APP slides if all were performed in-house*	*€56 (32–60*,* 16–80)*
Other immunohistochemical slides, external laboratory	€0 (0–0, 0–504)
Total cost	€624 (498–709, 268–1206)
* Total cost if all β-APP slides were obtained in-house*	*€94 (86–114*,* 40–598)*
Duration from autopsy to…	
Gross examination	0.7 months (0.5–1.0, 0.4–3.3)
Microscope slide examination	1.8 months (1.6–2.8, 1.0–4.0)
Completion of the consultation	2.3 months (1.6–3.0, 1.1–4.0)

β-APP = Beta-amyloid precursor protein, IQR = Interquartile range

### Sampling and stains

A median of 25 tissue samples were obtained per case (Table 1). All cases involved the examination of the brain (100.0%) and some additionally included sections of the spinal cord (12.0%). One case comprised an autolytic and poorly fixed brain that had already been cut fresh, which led to sparse sampling in this particular case. A median of seven β-APP slides were requested per case, whereas other IHC and special stains were clearly less frequent.

### Findings

Nearly all cases presented with macroscopic or microscopic haemorrhagic lesions in the brain parenchyma (88.0%; Table 1). Other common findings were oedema, hypoxic-ischaemic neuronal injury (mostly mild and terminal), and β-APP positivity consistent with a vascular axonal injury pattern. A detailed breakdown of the findings will be published in the future.

### Costs and duration

The median total cost of a pilot case was estimated to be €624 altogether (Table 1). Most of the total cost (median €588 per case) was made up of β-APP stains purchased from an external laboratory, as the in-house laboratory had not yet implemented β-APP into its services. Of note is the fact that if β-APP had been performed in-house, the median total cost of a pilot case would have been reduced to €94 altogether.

The median time from autopsy to gross examination (representing formaldehyde-fixation time) was 0.7 months, i.e., approximately three weeks. Median duration from autopsy to completion of the consultation was 2.3 months.

## Discussion

### Main findings

This paper introduced a Finnish in-house forensic neuropathology consultation pilot and its first 25 cases. On average one case was referred to consultation every 1—2 weeks. The cases involved mostly male decedents with TBI. The median total cost of an in-house pilot case was €624 per case, which is substantially lower compared to the previous outsourced practice (€1013 per case + €38/84 for each microscope slide with special/IHC stain, respectively). After the implementation of β-APP stain into the in-house laboratory service, the median total cost of a pilot case was reduced further to €94. The median duration of an in-house consultation was 2.3 months, which was of similar magnitude to the previous outsourced practice.

### First year experiences

The pilot cases have appeared to fit the in-house team’s expertise well in terms of consultation themes and degree of difficulty. Over the recent years, cases have been referred to a neuropathologist’s consultation relatively rarely at the Helsinki office [5]; this implies that forensic pathologists have been quite used to interpreting TBI findings themselves. In the pilot, consultation questions were often clear and well defined, and the neuropathology report was perceived as one additional piece of information to the forensic pathologist. Delightfully, several forensic pathologists participated in the gross examination and showed interest in developing their own skills in it. The in-house team have attempted to organize opportunities for this whenever possible.

Diagnostic problems have been minor. Typical challenging situations have concerned the interpretation of β-APP stain in cases with a short interval from injury to death, and in cases where a traumatic axonal injury may have been masked by an extensive vascular one. Other challenging cases have addressed epilepsy-related findings in the hippocampus, for example. When needed, external support has been sought and received from a board-certified hospital neuropathologist, on average once every 1—2 months. The aim has been to perform in-house gross examination and sampling in a similar manner to the external neuropathologist to facilitate proficient consultations.

The number of pilot cases has appeared appropriate relative to the available resources. The first half of the pilot year was somewhat busier than the second half. It should be noted, however, that an average case takes an estimated 3–4 h of the consultant’s working time, which is away from routine autopsy work. For example, the completion of two in-house consultation cases per week takes up a full working day’s worth of time.

Swift cooperation between in-house personnel (e.g., team leaders, forensic pathologists, autopsy technicians, laboratory scientists, and assistants) has been of primary importance for the pilot. In particular, the effort put into the implementation of β-APP into the in-house laboratory’s range of services has been significant. Delightfully, the pilot has revealed a wide range of know-how among the personnel of the office – an apt example being the database that was purpose-built without additional cost to the unit. Moreover, an assistant with a background in brain research has been of great help in recording the findings during gross examination.

### Future plans

As the routine operations of medico-legal cause-of-death investigation are financed from public funds, it is necessary to observe the cost aspect closely. Our approximate calculations based on the first-year data suggest that the median costs of an in-house consultation are markedly lower than those of an outsourced one. For example, if all the 25 first-year cases had been referred to an external provider, then the costs would have been over €25,000 higher; this calculation does not take into account IHC or special stains that would have been added to the costs. Of particular note is the fact that an in-house β-APP slide had over 90% lower cost compared to one purchased from the external provider. Over the recent years, there have been on average 30 cases referred to external consultation annually [5], about half of which would have matched the inclusion criteria of the in-house pilot. As such, the cost perspective clearly favours the option of performing as much of the examination and laboratory work in-house as possible. Not only would this reduce “brain drain” away from our institution, but also speed up the process and minimize the number of valuable tissue samples ending up in the archives of external institutions.

The inclusion criteria for cases accepted for in-house consultation could be gradually expanded in the future. On top of TBI and hypoxic-ischaemic encephalopathy, it may be possible to offer in-house consultations for cases involving certain neuropathological entities such as common neurodegenerative diseases and epilepsy-related deaths; these would be performed at the level that is sufficient for medico-legal cause-of-death investigation. In contrast, a wide range of other entities (e.g., paediatric cases, CNS tumours, autoimmune and metabolic diseases) will likely be referred to an expert hospital neuropathologist also in the future. Another future development goal will be the expansion of the in-house consultation practice to cover not only the Helsinki office but other regional offices of the Forensic Medicine Unit as well. This could be relatively easily achieved, as numerous types of samples are already transported between offices on a regular basis.

An imperative future goal is to actively and continuously expand the in-house team’s knowledge and practical skills. If funding makes it possible, the aim is to send team members to courses with different emphases (e.g., TBI, epilepsy, neurodegeneration) such that each member will build a unique competence profile. International networking would also be essential. As for providing education to forensic pathology residents, general forensic pathologists, and other professionals, the team have so far held presentations at local training events and given lectures to medical students. They have also started to compile an electronic collection of illustrative findings for potential educational use.

### Research possibilities

Even though it is essential to intertwine operational development with academic research, most effort of the in-house team has been directed to practical matters and implementation at the pilot stage. However, the team have attempted to consider potential future research perspectives as comprehensively as possible when planning the protocol. Assuming that cases will accumulate over the next few years as is expected, the consultation practice may be able to facilitate several research projects in the fields of forensic and translational neuropathology already in near future.

Studies aiming to utilize register-based data will likely benefit from the project. A potential draw-back of conventional open-ended pathology reports is the lack of uniformly reported details and potential difficulties in interpretation. Here, although somewhat time-consuming, the data on samples and findings are recorded in a detailed manner and mainly structured form. Data on macroscopic findings such as contusions include size, anatomical location, and specific cerebral sections to which the lesion extends. Microscopic findings such as β-APP positivity and microscopic haemorrhages are linked to specific tissue blocks. It is therefore easy to trace and review the findings later if needed. It is also possible to link the neuropathological variables to a wide range of other data on the cases.

Studies aiming to utilize CNS tissue material per se will also likely benefit from the project. The collection of samples has been systematic and of uniform quality. An average of 25 blocks per case have been collected according to a precise protocol. For example, the protocol includes a compact “screening block” for common degenerative diseases, which reduces the number of blocks required to undergo IHC staining in a screening scenario. Conventional blocks from relevant brain regions are also obtained and can be utilized in more accurate diagnostics if needed. It should be noted that our cases are medico-legal, many dying of TBI, so our samples could play a role as reference material for hospital cases. As with the routine cause-of-death investigation practice, all microscope slides and paraffin blocks are archived for potential future use.

### Limitations

This study had limitations to acknowledge. First, the pilot cases did not comprise a consecutive series. It is likely that some cases that would have met our inclusion criteria were referred to an external consultant instead of an in-house consultation. Unfortunately, data on cases referred to the external provider during the pilot year are not available yet. Second, the pilot cases were not systematically subjected to an external review to confirm the findings. However, the in-house team were trained to examine TBI and hypoxic-ischaemic encephalopathy cases independently, and had the possibility to consult a board-certified neuropathologist at a low threshold. Third, our cost calculations were approximate. Costs were defined here as additional expenses for the unit resulting from the consultation practice; these included identifiable material costs but not personnel costs, for example, as they would have remained unchanged regardless of the pilot. It should be noted that each consultation took several hours of an in-house consultant’s working time, and this aspect was not included in our approximate cost calculations. Finally, even though forensic neuropathology consultations can be organized in very different ways, there is a paucity of previous data on the usage, benefit, and costs of neuropathology consultations in medico-legal autopsies [5]. This impedes comparing our experiences with previous ones.

## Conclusions

In July 2023, an in-house neuropathology consultation pilot was established at the largest regional office of the Finnish forensic medicine authority as an alternative to the previous practice of full outsourcing. Thanks to a favourable in-house atmosphere towards the pilot, the implementation has taken place successfully, and the first year experiences are encouraging. The next steps would be a controlled expansion of the pilot’s inclusion criteria and the in-house laboratory’s IHC selection, as well as further training of the in-house expert team. While the costs of the in-house consultation practice appear to be markedly lower than those of an external provider, both alternatives should be available for cases where sufficient expertise cannot be found in-house. Hopefully, the data and sample material accumulated as part of the in-house consultation practice will facilitate future research projects in the fields of forensic and translational neuropathology.

## Supplementary Information

Below is the link to the electronic supplementary material.

ESM 1 (DOCX 35.8 KB)

## Data Availability

The data underlying this article cannot be shared publicly due to local privacy regulations.
