# Supplementary material for: First year of in-house forensic neuropathology consultations in Helsinki, Finland
Source: Int J Legal Med. 2025 Jan 13;139(2):805–15. doi: 10.1007/s00414-024-03399-6 (PMC11850508; doi:10.1007/s00414-024-03399-6)
Supplement: Supplementary file 1 — (DOCX 35.8 KB) [file 414_2024_3399_MOESM1_ESM.docx]

SUPPLEMENTARY TABLES

**Supplementary Table 1**. Routine sampling protocol.

| Anatomical location | Specification |
| --- | --- |
| Cerebral cortex |  |
| Frontal cortex | To include anterior watershed |
| Temporal cortex | To include superior and middle gyrus |
| Parietal cortex | To include posterior watershed |
| Occipital cortex | To include calcarine sulcus |
| Cingulate cortex | Anterior |
| Other cerebral structures |  |
| Frontal white matter | Parasagittal |
| Corpus callosum, genu |  |
| Corpus callosum, splenium |  |
| Caudate nucleus | To include anterior limb of the internal capsule |
| Lenticular nucleus | To include insular cortex |
| Thalamus, right | To include posterior limb of the internal capsule |
| Thalamus, left | As above |
| Hippocampus, right | To include cornu Ammonis, parahippocampal gyrus, and fusiform gyrus |
| Hippocampus, left | As above |
| Basal forebrain | To include amygdala and commissura anterior |
| Brainstem |  |
| Midbrain | To include substantia nigra |
| Rostral pons | Rostral to the superior cerebellar peduncles |
| Caudal pons, right | To include middle cerebellar peduncles |
| Caudal pons, left | As above |
| Medulla oblongata | To include inferior olivary nuclei |
| Cerebellum |  |
| Cerebellum, hemisphere | To include cerebellar cortex and dentate nucleus |
| Cerebellum, vermis |  |
| Screening block for common degenerative diseases | Small sections of the following: - Cornu Ammonis and parahippocampal gyrus on either side  - Frontal cortex  - Calcarine cortex - Lenticular nucleus - Substantia nigra - Cerebellar cortex and dentate nucleus |

**Supplementary Table 2**. Documentation of cases in the electronic database.

| Variable | Specification |
| --- | --- |
| Metadata |  |
| Case number |  |
| Name of in-house consultant | The person responsible for the case |
| Other consultants | Both external and internal consultants |
| Status of the consultation | Gross examination/Microscopy/Completed |
| Time stamps for status changes | Dates |
| Background information |  |
| Brain weight | Fresh/After formaldehyde fixation |
| Samples and stains |  |
| Cassette number |  |
| Anatomical location | Selected from a structured list |
| Slice (cerebral samples only) | Anterior (A)/Posterior (P) + ordinal number denoting the section from the mamillary bodies, e.g., “A1” or “P4” |
| Stains | Selected from a structured list |
| Additional details | Open-ended field |
| Total number of samples |  |
| Findings |  |
| Anatomical location | Selected from a structured list |
| Slice (cerebral samples only) | As above |
| Finding/diagnosis | Selected from a structured list |
| Severity | Mild/Moderate/Severe/Undetermined/Other |
| Age | Acute/Subacute/Chronic/Acute-on-chronic/Sequelae/Undetermined/Other |
| Size (macroscopic findings only) | Maximum perpendicular dimensions |
| Cassette number (microscopic findings only) |  |
| Stains (microscopic findings only) | Selected from a structured list |
| Additional details | Open-ended field |
| Total number of findings |  |
| Report |  |
| Summary and conclusion | Open-ended field |
| Signature of the in-house consultant | Electronic signature |
